# Evaluator’s alignment as an important indicator of adequacy of the criteria and assessment procedure for recognizing the good practice in public health

**DOI:** 10.3389/fpubh.2024.1286509

**Published:** 2024-04-22

**Authors:** Matej Vinko, Tina Lesnik, Sandra Radoš Krnel

**Affiliations:** National Institute of Public Health, Ljubljana, Slovenia

**Keywords:** criteria, evidence-based interventions, public health practice, public health interventions, good practice portals

## Abstract

**Background:**

Public health interventions aim to reduce the burden of chronic non-communicable diseases. Implementing evidence-based interventions that are proven to be successful and effective is widely recognized as the best approach to addressing public health challenges. To avoid the development and implementation of less effective or successful or even harmful practices, clear criteria for the assessment of practices, that consider different dimensions of the interventions in public health, are needed. The main aim of the research was to test our Criteria and assessment procedure for recognizing good practices in the field of public health by estimating the consistency between the evaluators and thereby gaining insight into the adequacy and reliability of the criteria as well as to check how the evaluators understand the criteria and methodology and if it is properly used in assessing the interventions.

**Methods:**

The assessment of the interventions took place from 2021 to 2022. The individual evaluator’s scores on the scale from 1 to 5 for each specific sub-criterion were collected, which was followed by a panel discussion to reach a final score for each sub-criterion. The inter-rater agreement was measured using percent overall agreement and Fleiss’ kappa coefficient.

**Results:**

We found moderate inter-rater agreement on the level of the assessment criteria group. The lowest agreement was observed for the effectiveness and efficiency sub-criteria group, which also received the lowest scores from the evaluators. Challenges identified with the scoring process were due to the descriptive 1 to 5 scale and the varying specificity of the criteria.

**Conclusion:**

The results showed that studying consistency between evaluators can highlight areas for improvement or adjustment in the assessment criteria and enhance the quality of the assessment instrument. Therefore, such analysis would be useful part of both newly and well-established health promotion and prevention program registries.

## 1 Background

Public health interventions aim to reduce the burden of chronic non-communicable diseases by addressing risk factors such as tobacco smoking, alcohol consumption, unhealthy diet, physical inactivity, and overweight (1). Implementing evidence-based interventions that are proven to be successful and effective in improving individual, community, and population health is widely recognized as the best approach to addressing public health challenges (2, 3). While randomized controlled trials held as gold standard for the quality of evidence that supports causality between the intervention and outcomes, there is growing awareness of the importance of demonstrating effectiveness in actual program settings for public health interventions (4–6). Practice-based evidence, including theories and approaches such as community-based participatory research, PRECEDE-PROCEDE, and RE-AIM framework, has been proposed as a more relevant source of evidence for public health decision-making due to the focus on populations, consideration of contextual factors, and complexity of multi-disciplinary interventions (4, 7, 8). Therefore, evaluation of existing public health interventions and selection of best practices are a valuable source of practice-based evidence (4, 9, 10). To avoid the development and implementation of less effective or successful or even harmful practices, clear criteria for the assessment of practices, that are considering different dimensions of the interventions in public health, are needed (11). Health promotion and prevention program registries (HPPRs) serve as valuable “portals for the exchange of good practices” as long as appropriate evaluation and assessment criteria are utilized when selecting the presented practices. These registers increase transparency and highlight effective and successful interventions, aiding decision-makers in selecting and implementing the most appropriate interventions. They serve as entry points and practice repositories, providing easy access to evidence-based practices (12, 13). There is a number of practice portals within the health domain in the EU, such as EU Best Practice portal, The European Monitoring Center for Drugs and Drug Addiction (EMCDDA) portal, Healthy Workplaces Campaigns of good practice, and several national best practice portals. This is a welcome development as it means that more institutions have recognized the need and added value of this approach. The exchange of best practices has a potential to improve health by demonstrating what interventions worked well in similar settings and populations, and it avoids “re-inventing the wheel” in designing and piloting similar interventions, building upon ones’ expertise and more efficient use of resources (14).

Nevertheless, the challenge of choosing the “right” approach and criteria in assessing the health promotion and disease prevention interventions still remains (15, 16). This was recognized as one of the important challenges at the EuroHealthNet Thematic Working Group on health promotion and disease prevention program registries (17). The development of the system for the assessment of health promotion and disease prevention interventions consists of several steps, from defining the criteria, development of evaluation methodology, selection of evaluators, piloting the assessment procedure, and, finally, regular use of the entire system/portal. An often-overlooked but important step is how the evaluators understand the criteria and methodology if it is properly used in assessing the interventions (18).

The main aim of our research was to test our criteria and assessment procedure for recognizing good practices in the field of public health by estimating the consistency between the evaluators, thereby gaining insight into the adequacy and reliability of the criteria as a measuring instrument for the assessment of the interventions and to check how the evaluators understand the criteria and methodology if it is properly used in assessing the interventions.

## 2 Methods

### 2.1 Assessment criteria for evidence-based public health interventions

The Slovenian “criteria for assessing public health interventions for the purpose of identifying and selecting good practices” were developed based on the European Commission’s Criteria to select best practices in health promotion and disease prevention and management in Europe (14, 19). The major difference between these criteria is that European Commission’s Criteria are focusing on selecting “best” practices while the Slovenian Criteria are intended to acquire also those practices that are recognized as examples of “good” practices and have a potential to further develop and improve.

The aim of the Slovenian criteria is to establish a system for recognizing examples of good practices and promote the use of these approaches in the field of public health. The objectives of the Slovenian HPPRs are (1): to raise the standards of public health interventions and improve their quality (2); to provide an overview on quality and effectiveness of public health interventions; and (3) to support knowledge exchange and the use of effective approaches by providing a pool of reviewed interventions.

**Figure 1:**
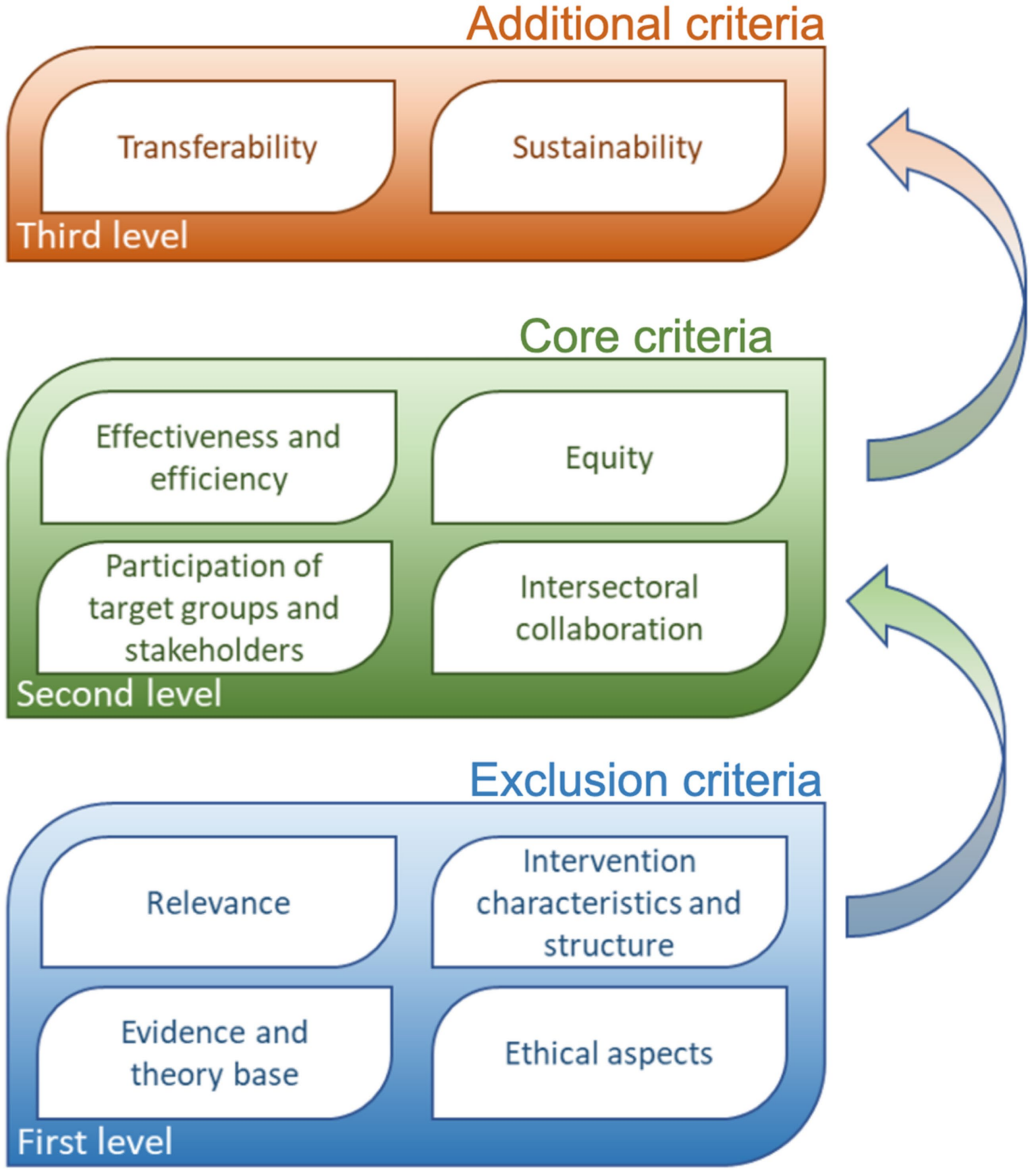
Criteria for assessing public health interventions for the purpose of identifying and selecting good practices.

The criteria are organized in three levels, namely, exclusion, core, and additional criteria (Figure 1), and each group of criteria is used in successive manner to assess submitted interventions. The exclusion criteria assess the adequacy and completeness of the information provided and whether the intervention meets the basic conditions for further assessment. It is the first sieve, where it is assessed whether the intervention has a political and strategic relevance, is supporting current public health needs, furthermore these criteria are assessing if the intervention has a potential to produce beneficial results for the population in need in a scientifically sound manner, is free from any commercial benefits and have key elements for being successful or there is a risk that it could be harmful, unjust or ineffective. An intervention that passes the first inclusion threshold is further evaluated according to the core criteria that include its effectiveness and efficacy, as well as its contribution to reducing health inequalities. At the third level, the potential to transfer the intervention to other areas, another geographical environment, and another population is assessed. Therefore, additional criteria include an assessment of whether the interventions contain elements that enable the adaptation, upgrade, or transfer of the intervention to other settings. As recommended by many scholars, the Slovenian Criteria included the key elements for the assessment of public health interventions such as importance of assessing the implementation process and short-term and long-term outcomes, influence of contextual factors, importance of setting the objectives, theoretical underpinnings, and scope of interventions, and issues of sustainability, relevance, and stakeholder collaboration (4, 16, 20–26).

For the purpose of assessment, each sub-criterion is assigned one of the numerical values (from 1–the intervention does not meet the requirements or does not take into account the criterion being studied or cannot be assessed due to missing or incomplete information to 5–the intervention successfully addresses all important aspects of the assessment criteria.), with the exception of the group of criteria used to evaluate the ethics of the intervention, where only yes or no answers were possible.

### 2.2 Data and processes

At least three public health professionals independently evaluated five interventions using the assessment criteria for evidence-based public health interventions (two interventions were evaluated by four evaluators and three interventions were evaluated by three evaluators). One evaluator was a medical doctor and an expert in the priority public health area that the interventions were addressing alcohol use. Second and third evaluators were medical doctors and experts in public mental health and epidemiology of non-communicable diseases. Fourth evaluator was a psychologist. The evaluators were familiar with each other, either due to working on the development of the Slovenian criteria or other public health research-related projects within the public health institute where they were all employed. The team of evaluators was selected on a personal invitation based on the leading expert of the team that developed Slovenian Criteria which also acted as one of the evaluators. Each intervention was evaluated by assigning a numerical value (from 1 to 5) to each criterion. The assessment took place from February 2021 to June 2022. Individual scores of the assessment criteria for each intervention were compiled, and a panel discussion was held to reach a consensus on the final score.

### 2.3 Statistical analysis

To determine the inter-rater agreement percent overall agreement (POA), Fleiss’ kappa (FK) coefficient with 95% confidence intervals and standard error were estimated (27). We assessed the inter-rater agreement on the level of the criteria for assessing public health interventions and the level of individual interventions that were included in the pilot assessment process. Values from 1.00 to 0.81 were described as high agreement, 0.80 to 0.61 were described as substantial agreement, 0.60 to 0.41 were described as moderate agreement, 0.40 to 0.21 were described as fair agreement, 0.20 to 0.00 were described as slight agreement, and values below 0.00 were described as poor agreement (28). Additionally, we provide average scores by individual evaluators (AS), final score (FS) reached, and the difference in scores between AS and FS (delta). To assess the correlation between average scores by individual evaluators and Fleiss’ kappa coefficient, Spearman’s Rho was calculated.

## 3 Results

Inter-rater agreement on the level of the assessment criteria group for evidence-based public health interventions was moderate (FK = 0.43 (0.36–0.49), SE = 0.0004) (54.1%) for overall agreement.

The highest inter-rater agreement on the level of exclusion criteria was achieved among the relevance sub-criteria group with all criteria rated 5 by all evaluators (Table 1). A moderate agreement was reached for the intervention characteristics and structure sub-criteria group, with slight agreement for criteria 2.8 to 2.11. The average FS and AS for this group was 3.9. Fair agreement was reached for the evidence and theory-based sub-criteria group, with an average FS of 4.2 and AS of 4.4.

**Table 1:** Inter-rater agreement and scoring values of the exclusion criteria.

	Exclusion criteria								
		POA	FK	0.05 CI	0.95 CI	SE	AS	FS	Delta
1. Relevance		100%	1	1	1	0.00	5	5	0
1.1.	The intervention is compliant with public health strategy on a local, regional, national, or European level.	100%	1	1	1	0.00	5	5	0
1.2.	The intervention is put in place to support the implementation of strategic documents, legislation, or other initiatives addressing the priority public health area.	100%	1	1	1	0.00	5	5	0
		POA	FK	0.05 CI	0.95 CI	SE	AS	FS	Delta
2. Intervention characteristics and structure		52,7%	0,41	0.28	0.54	0.02	3.9	3.9	0
2.1.	Situational analysis of the priority public health area addressed by the intervention is clearly presented (e.g., SWOT analysis) and based on a well-defined methodology of primary or secondary data collection and analysis.	60%	0.5	0.1	0.9	0.18	4.1	4	−0.1
2.2.	Description of the target population is sufficiently segmented (by demographic, psychographic, behavioral, and other relevant factors).	87%	0.83	0.51	1	0.11	4.1	4	−0.1
2.3.	The choice of the target population(s) is clearly described.	73%	0.67	0.27	1	0.16	4.4	5	0.6
2.4.	Behavioral and/or communication objectives are defined using SMART principle.	47%	0.33	−0.22	0.89	0.25	3.7	3.4	−0.3
2.5.	Strategies, tactics, and tools used to reach behavioral and/or communication objectives are described.	87%	0.83	0.51	1	0.11	4.9	4.8	−0.1
2.6.	The indicators for process and outcome evaluation are described.	47%	0.33	−0.22	0.89	0.25	3.3	3	−0.3
2.7.	The contribution of the target population, carers, health professionals, and/or other stakeholders as applicable was appropriately planned, supported, and resourced.	73%	0.67	0.27	1	0.16	4.3	4.4	0.1
2.8.	The intervention includes an adequate estimation of the resources (human resources, financial resources etc.) in clear relation with committed tasks (development, implementation, and evaluation).	20%	0	−0.2	0.2	0.09	3.5	3.6	0.1
2.9.	Information on the optimization of resources for achieving the objectives is provided (planned and actual costs of the intervention).	33%	0.17	−0.28	0.61	0.20	3.1	3.6	0.5
2.10.	Evaluation is structured and described on three levels: formative evaluation, process evaluation, and evaluation of effectiveness and/or efficiency.	27%	0.08	−0.08	0.25	0.07	3.5	3.4	−0.1
2.11.	The documentation (guidelines, protocols, etc.) supporting the intervention is presented properly and referenced throughout the text.	27%	0.08	−0.08	0.25	0.07	3.5	3.2	−0.3
		POA	FK	0.05 CI	0.95 CI	SE	AS	FS	Delta
3. Evidence and theory base		47%	0,33	0.03	0.64	0.10	4.4	4.2	0.17
3.1.	The intervention is built on a well-founded theory and is evidence-based.	60%	0.5	0.1	0.9	0.18	4.5	4.4	−0.1
3.2.	The effective elements (or techniques or principles) in the approach are stated and/or justified.	33%	0.17	−0.28	0.61	0.20	4.2	4	−0.2

Among the core criteria group, the inter-rater agreement on the level of effectiveness and efficiency sub-criteria group was only slight (Table 2). Average FS in the sub-criteria group was 3.4 and the average AS was 3.2, which were the lowest scores of all sub-criteria groups. Inter-rater agreements of the equity, participation of target groups and stakeholders, and intersectoral collaboration sub-criteria groups were substantial or moderate with all reaching AS and FS of 4 or higher.

**Table 2:** Inter-rater agreement and scoring values of the core criteria.

	Core criteria								
		POA	FK	0.05 CI	0.95 CI	SE	AS	FS	Delta
1. Effectiveness and efficiency		35%	0,19	0.06	0.32	0.02	3.2	3.4	0.2
1.1.	The intervention has undergone process evaluation (internal or external) which was well described.	47%	0.33	0.01	0.66	0.15	3.7	3.8	0.1
1.2.	The evaluation outcomes (e.g., number of participants, satisfaction with the intervention, and media coverage) were linked to the stated goals of the intervention.	40%	0.25	−0.15	0.65	0.18	3.4	3.8	0.4
1.3.	The evaluation outcomes are relevant given the type of the intervention, theoretical base of the intervention, and the target population.	20%	0	−0.2	0.2	0.09	3.1	3.5	0.4
1.4.	An evaluation study has been performed (based on needs and challenges) between the initial and final situation and is accounting for possible biases.	47%	0.33	−0.22	0.89	0.25	3.1	3.3	0.2
1.5.	All improvements in comparison to the starting point, for example, the baseline concerning outcomes in different areas are documented and presented.	53%	0.42	−0.07	0.91	0.22	3.2	3.3	0.1
1.6.	The intervention has been evaluated from an economic point of view.	33%	0.17	0.17	0.17	0.00	2.7	3.3	0.6
1.7.	The evaluation outcomes demonstrated beneficial impact.	20%	0	−0.2	0.2	0.09	3.1	2.8	−0.3
1.8.	Possible negative effects have been identified and stated.	20%	0	−0.49	0.49	0.22	3.3	3.8	0.5
		POA	FK	0.05 CI	0.95 CI	SE	AS	FS	Delta
2. Equity		58%	0,47	0.2	0.74	0.07	4	4.4	0.42
2.1.	The relevant dimensions of equity are adequately and actively considered throughout the process of implementing the intervention (e.g., age, gender, socioeconomic status, rural and/or urban area, and vulnerable groups).	27%	0.08	−0.08	0.25	0.07	4	4.5	0.5
2.2.	Efforts to identify vulnerable population groups, inequities in access to the intervention and other possible manifestations of inequities are evident from the intervention plan and are documented in the intervention documentation (guidelines, recommendations etc.).	47%	0.33	−0.22	0.89	0.25	3	3.8	0.8
2.3	The intervention includes actions to empower target population group (e.g., increasing health literacy, social support, and decision-making skills).	100%	1	1	1	0.00	5	5	0
		POA	FK	0.05 CI	0.95 CI	SE	AS	FS	Delta
3. Participation of target groups and stakeholders		73%	0,67	0.4	0.93	0.08	4.6	4.63	0.03
3.1.	The structure, organization, and content (also evaluation outcomes and monitoring) of the intervention were defined and established together with the relevant stakeholders and the target population.	87%	0.83	0.51	1	0.11	4.7	4.8	0.1
3.2.	Mechanisms facilitating sustainable participation of several agents involved in different stages of the intervention (development, implementation, and evaluation) have been established and are well described.	60%	0.5	0.1	0.9	0.18	4.5	4.5	0
		POA	FK	0.05 CI	0.95 CI	SE	AS	FS	Delta
4. Intersectoral collaboration		64%	0,56	0.34	0.77	0.06	4.3	4.3	0
4.1.	Several sectors collaborated to carry-out the intervention.	73%	0.67	0.27	1	0.16	4.4	4.8	0.4
4.2.	The intervention was developed through interdisciplinary collaboration and is supported by relevant stakeholders (e.g., health and social care professionals at all levels, civil society, public institutions from education, employment, and digital services).	73%	0.67	0.27	1	0.16	4.4	4	−0.4
4.3.	The intervention promotes coordination among several sectors (e.g., health, social, and education).	47%	0.33	0.01	0.66	0.15	4.2	4.3	0.1

In the additional criteria group, the inter-rater agreement for the transferability sub-criteria group was moderate with an average FS of 3.9 and AS of 3.8 (Table 3). The agreement for the sustainability sub-criteria group was fair, with an average FS of 4.3 and AS of 3.5, which also had the largest difference between FS and AS among all sub-criteria groups.

**Table 3:** Inter-rater agreement and scoring values of the additional criteria.

	Additional criteria								
		POA	FK	0.05 CI	0.95 CI	SE	AS	FS	Delta
1. Transferability		61%	0,51	0.34	0.68	0.03	3.8	3.9	0.1
1.1.	The intervention uses instruments (e.g., manual with a detailed activity description) that allow for repetition/transfer.	60%	0.5	0.1	0.9	0.18	3.8	3.3	−0.5
1.2.	The description of the intervention includes all organizational elements identifies the limits and necessary actions that were taken to overcome legal, managerial, or financial barriers.	47%	0.33	0.01	0.66	0.15	2.9	2.8	−0.1
1.3.	The description includes all contextual elements of the beneficiaries (demographic, psychographic, attitudinal, and other characteristics) and the actions that were taken to overcome personal and environmental barriers.	60%	0.5	0.1	0.9	0.18	3.7	4.3	0.6
1.4.	A communication strategy and a plan to disseminate the results have been developed and Implemented.	67%	0.58	0.07	1	0.21	3.7	4	0.3
1.5.	The intervention has been successfully transferred/repeated from a pilot setting into a larger-scale area.	87%	0.83	0.51	1	0.11	4.3	4.8	0.5
1.6.	The intervention shows adaptability to different contexts and challenges encountered during its implementation.	47%	0.33	−0.22	0.89	0.25	4.2	4.5	0.3
		POA	FK	0.05 CI	0.95 CI	SE	AS	FS	Delta
2. Sustainability		48%	0,35	0.18	0.52	0.03	3.5	4.3	0.7
2.1.	The intervention has institutional support, an organizational and technological structure and stable human resources.	47%	0.33	0.01	0.66	0.15	4.1	4.5	0.4
2.2.	The intervention presents a justifying economic report, which also discloses the sources of financing.	60%	0.5	0.1	0.9	0.18	3.7	4.3	0.6
2.3.	The relevant stakeholders or communities have ensured the continuation of the intervention through institutional anchoring and/or ownership in the medium and long term.	27%	0.08	−0.08	0.25	0.07	3.4	3.5	0.1
2.4.	The intervention provides training of staff in terms of knowledge, techniques, and approaches in order to sustain it.	60%	0.5	0.1	0.9	0.18	3.9	4.3	0.4
2.5.	A sustainability strategy has been developed that considers a range of contextual factors (e.g., health and social policies, innovation, cultural trends and general economy, and epidemiological trends).	47%	0.33	−0.22	0.89	0.25	2.5	4.8	2.3

**Figure 2:**
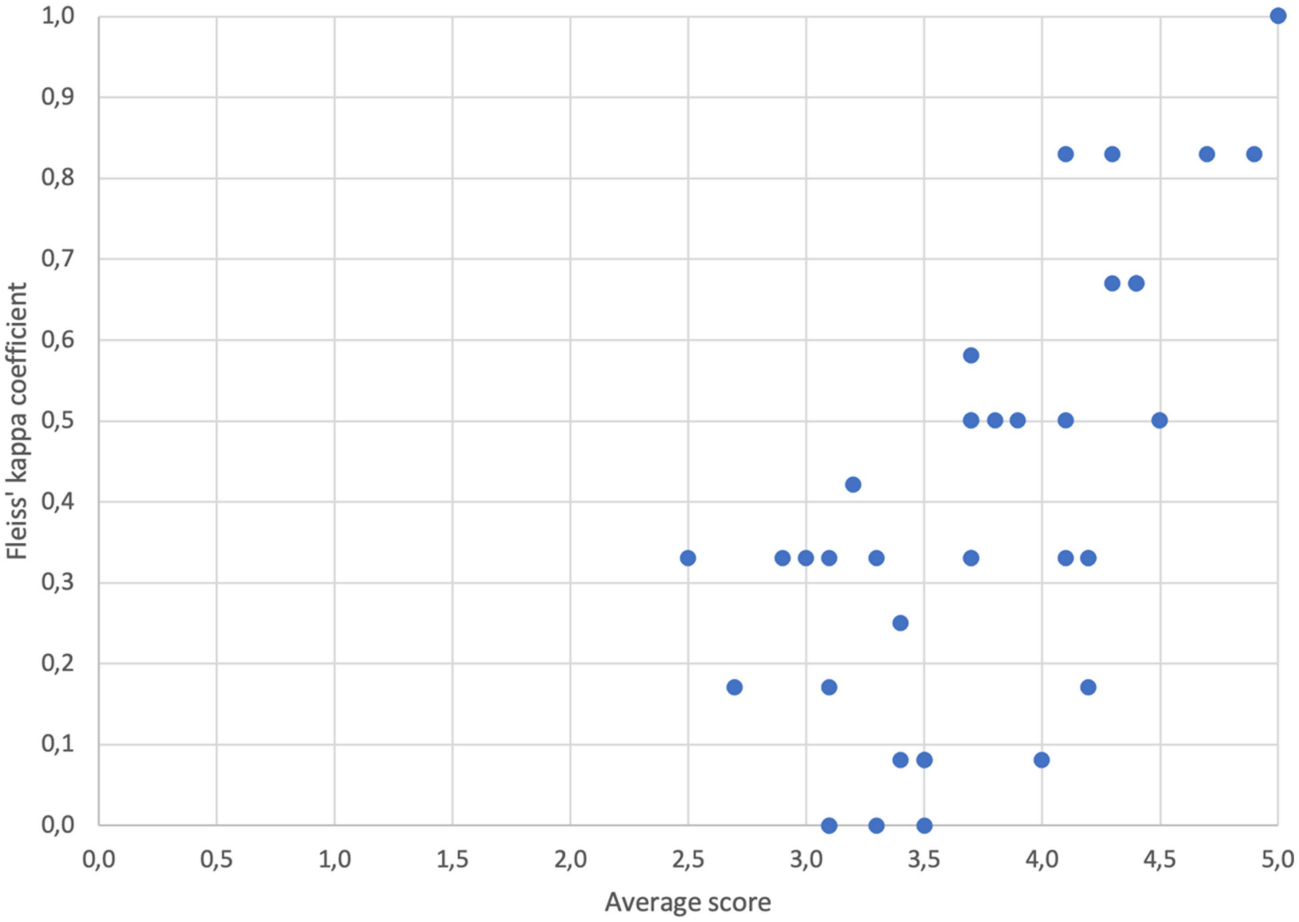
Association between average scores by individual evaluators and Fleiss’ kappa coefficient (r_s_ = 0.73577, *p* < 0.0001).

A statistically significant correlation (r_s_ = 0.73577, *p* < 0.0001) is present on the level of AS and FK with criteria with higher AS reaching higher inter-rater agreement and vice versa (Figure 2).

## 4 Discussion

The primary purpose of an intervention assessment for recognizing the good practice in public health is to impact decision-making. The level of intricacy and accuracy required in the evaluation is contingent on the needs of the decision maker and the nature of decisions that will be made based on the results (29). The results presented in this article showed a moderately high degree of consistency in the assessment, demonstrating the validity of the Slovenian Criteria as a useful tool for identifying and promoting effective public health interventions. Despite being the first time that the criteria were used and the evaluators lacking prior experience, a moderate level of inter-rater agreement was achieved. As part of the comprehensive assessment, a concurrent review and updating of criteria was performed, resulting in the establishment and optimization of the assessment procedure.

The lowest agreement was observed for the effectiveness and efficiency of the sub-criteria group, which also received the lowest scores from the evaluators. A relationship between low scores and low inter-rater agreement was noted among the criteria. This could be attributed to the scoring method, where criteria were rated on a scale of 1 to 5 with descriptions for each grade. The grade 3 is described as “the intervention generally addresses this criterion well, with few shortcomings remaining.” The evaluators relied on these descriptions to score the interventions, but when interventions performed poorly, the evaluators had to determine the magnitude of shortcomings and score accordingly, leading to subjectivity. Additionally, criteria with varying specificity caused challenges in assigning scores, as the evaluator had to make subjective assessments of the contribution of individual processes or aspects to the final score. For example, criterion 1.3 in the core criteria group (“the evaluation outcomes are relevant given the type of the intervention, theoretical base of the intervention and the target population”) required the evaluator to provide a single score for three different but related aspects, which added complexity to the scoring process. The issue of subjectivity is probably common problem of health promotion and prevention program registries (HPPRs) since most European national HPPRs have developed assessment criteria divided into three to four main assessment sections and multiple sub-sections and are using scoring system that requires from the evaluator to determine how successful is the intervention in fulfilling the criteria and score accordingly (12, 18). In fact, some degree of subjective judgments is unavoidable in any evaluation, for instance, in weighing the importance of the various criteria used (30). In addition, Ng and De Colombani in their systematic literature review found out that the subjectivity at various stages of selection or evaluation is a universal feature across all reviewed sources (4).

The interventions were assessed using a questionnaire and supplementary intervention documentation such as guidelines and evaluation studies supplied by the owners of the interventions. The completeness and organization of the literature, however, varied greatly among the interventions, and some parts of the questionnaire were narrative and qualitative to accommodate the uniqueness of the practice, which could make it difficult for evaluators to extract the relevant information for scoring. To generate appropriate evidence for effective interventions, it is vital to adhere to the basic principles of evidence-based public health, which necessitate comprehensive intervention documentation (8, 31). Providing in-depth guidance on how to effectively present documentation before the assessments could greatly enhance the usability and effectiveness of the tool. This added level of detail can also help streamline the assessment process and make it simpler for users to understand and implement.

Similar methodological approaches are used in prevention programs that take place in a clinical setting confirming its usefulness in supporting decision-making process. For example, in breast cancer screening programs, radiologists perform a third independent reading in cases of disagreement between the first two independent readings, and the inter-rater agreement is then calculated (32, 33).

A limitation of our analysis is the choice of the inter-rater agreement measure we used (34). Since we did not use weighted Fleiss’ kappa coefficient or any other measure that consider the distance in the evaluation of inter-rater agreement, the magnitude of disagreement between raters is not reflected in the computed Fleiss’ kappa value. Additionally, evaluators did not receive training on the use of the criteria. It is expected that the agreement between evaluators would have been higher if they had received training in the use of the assessment tool before assessing the pilot interventions. The lack of training may have resulted in inconsistent application of the tool and a lower level of agreement among evaluators (35). However, the evaluators experienced public health professionals and sufficiently proficient in all theoretical and practical domains described by the criteria. Careful consideration of the composition of the panel of reviewers is recognized as an important element of the assessment procedure to avoid biases due to vested interests, and details of the composition should be made transparent (4).

## 5 Conclusion

The development and use of criteria for the assessment of practices that are considering different dimensions of the interventions in public health offers valuable insights for various stakeholders into the realm of public health. It caters to funders or clients by presenting a clear and informative categorization of practices into “best” and “good” categories. Additionally, it benefits researchers and practitioners who are involved in the development and implementation of interventions by offering specific feedback on each criterion that can assist in further refining the practice.

Despite confirmed usefulness and the importance of best practice assessment instruments, there is a relative lack of research on their performance (4). Furthermore, the literature on evaluator’s agreement in assessment of the specific intervention, as an important indicator of reliability of assessment procedure, is scarce. In this study we have shown that the inter-rater agreement differs across the sub-criteria groups depending on clarity of descriptions of specific criterion and scoring system, especially for the interventions that performed poorly or that were not successful in fulfilling the requirements of specific criterion or group of criteria. This discovery prompted us to investigate these criteria further and make necessary adjustments to increase the reliability of the assessment process. Studying the consistency between evaluators can provide valuable insights into the performance of the assessment instrument. This is not just of great importance for the institutions that are currently in the phase of developing or just have developed criteria for the assessment of interventions in the field of prevention and health promotion but also for well-established HPPRs. Such analysis can reveal areas of the assessment or specific criteria that perform inadequately and need improvement or adjustment. Through this process, researchers can gather valuable information that can be used to enhance the overall quality of the assessment instrument. Improving the assessment and selection process of good/best practices can then facilitate and promote the use of the practice-based evidence which can complement research findings in public health. Further research is needed to clarify the importance and usefulness of the inter-evaluator alignment and the best methodology for determining it.

To further improve best practice assessments, we suggest involving policymakers more extensively in the assessment process. This could include their participation in either the development or upgradation of the criteria and during the actual assessment process (36). While researchers may prefer to maintain independence from policymaking and implementation, public health research can have the most significant impact when researchers, practitioners, and decision-makers take responsibility for its production and application (37).

## Data availability statement

The data analyzed in this study is subject to the following licenses/restrictions: The data is provided by the owners of the interventions included in the research. Requests to access these datasets should be directed to statisticna.pisarna@nijz.si.

## Author contributions

MV: Conceptualization, Data curation, Formal analysis, Investigation, Methodology, Validation, Writing – original draft, Writing – review & editing. TL: Conceptualization, Investigation, Methodology, Validation, Writing – review & editing. SR: Conceptualization, Methodology, Supervision, Validation, Writing – original draft, Writing – review & editing.
